# Progesterone Modulates Protein Synthesis in Osteoarthritic Fibroblast-like Synoviocytes

**DOI:** 10.3390/ijms27198899

**Published:** 2026-10-07

**Authors:** Fadi Askar, Georgios Chatziagorou, Sofia Wüstenhagen, Alessia Capraro, Björn Andersson, Evelin Berger, Daniela Esposito, Luminita Labusca, Carmen Corciulo

**Affiliations:** 1 Department of Pharmacology, Institute of Neuroscience and Physiology, University of Gothenburg, 41390 Gothenburg, Sweden; 2 SportsMed, Carlanderska Hospital, 40545 Gothenburg, Sweden; 3 Department of Orthopedics, Institute of Clinical Sciences, Sahlgrenska Academy, University of Gothenburg, 41390 Gothenburg, Sweden; 4 Department of Rheumatology and Inflammation, Institute of Medicine, Sahlgrenska Academy, University of Gothenburg, 41346 Gothenburg, Sweden; 5 Bioinformatics and Data Centre, Sahlgrenska Academy, University of Gothenburg, 41390 Gothenburg, Sweden; 6 Proteomics Core Facility, SciLifeLab, Sahlgrenska Academy, University of Gothenburg, 41390 Gothenburg, Sweden; 7 Department of Endocrinology, Sahlgrenska University Hospital, University of Gothenburg, 41345 Gothenburg, Sweden; 8 National Institute of Research and Development in Technical Physics, 700050 Iasi, Romania; 9 Orthopedics and Trauma Clinic, County Emergency Hospital, 700111 Iasi, Romania

**Keywords:** osteoarthritis, synoviocytes, progesterone

## Abstract

Knee osteoarthritis (OA) is highly prevalent in women and is characterized by structural joint alterations and synovial inflammatory processes that contribute to pain and impaired mobility. In a previous study, we identified a positive correlation between progesterone levels and pain intensity in postmenopausal women. However, the role of progesterone in synovial inflammation remains poorly understood. Therefore, this study aimed to characterize sex-specific differences in synovial tissue between postmenopausal women and age-matched men and to investigate the association between progesterone levels and the identified molecular profile. Whole blood and synovial tissue were collected from women and men with knee OA to assess plasma progesterone concentrations and the proteomic profiles of synovial tissue and isolated primary fibroblast-like synoviocytes (FLSs). The expression and localization of progesterone receptors were evaluated using RNAscope and immunofluorescence. FLS stimulated with physiological concentrations of progesterone were used for de novo protein synthesis assays. Quantitative proteomics of synovial tissue revealed enrichment of protein synthesis-related pathways in women compared to age-matched men, and isolated FLS from women with knee OA exhibited a pro-inflammatory and pro-fibrotic proteomic profile. Stratification by plasma progesterone levels identified two distinct patient groups, where lower progesterone levels were associated with pro-inflammatory signatures. The RNAscope analysis demonstrated increased transcriptional activity in synovial tissue from women with knee OA, localized to CD90^+^ sublining FLS. However, progesterone receptor expression at both the RNA and protein levels showed no sex-specific differences. Moreover, physiological concentrations of progesterone inhibited *de novo* protein synthesis in FLS isolated from men with knee OA. These findings underscore the hormonal context-dependent modulatory effects of progesterone on synovial inflammation and fibroblast activity. The study further highlights the importance of incorporating sex-specific hormonal profiles into OA patient stratification to support more precise and targeted therapeutic strategies.

## 1. Introduction

Osteoarthritis (OA) is a common degenerative joint disease characterized by the erosion of articular cartilage, alterations in the subchondral bone, and inflammation of the synovial membrane. It is strongly associated with aging and characterized by pain and reduced joint functionality, which are the most prominent clinical features of the disease [1,2]. Inflammation of the synovial membrane, synovitis, shows a stronger association with pain intensity, particularly in the context of nociceptive pain [3]. Sustained inflammation can, in addition, induce aberrant nerve sprouting, contributing to neuropathic pain [4]. Inflammation of the synovial tissue leads to the activation of fibroblast-like synoviocytes (FLSs), which further amplify the inflammatory response. Activated FLS secrete inflammatory mediators and matrix-degrading enzymes that promote cartilage and bone erosion, as well as excessive extracellular matrix components that contribute to tissue fibrosis, leading to reduced joint flexibility [5,6]. Studying the mechanisms of disease progression helps to move forward a relevant therapeutic strategy aiming to reduce inflammation and pain. This is made difficult by the heterogeneity of the OA population. Increasing efforts are now being directed toward identifying disease characteristics that can facilitate patient stratification [7]. For instance, pain and inflammation are not consistently present across all patients, and hormonal alterations in postmenopausal women may define a distinct subgroup within the OA population [8]. In general, inflammatory mechanisms are influenced by gonadal hormones [9], which may partly explain the higher pain intensity and disease prevalence observed in postmenopausal women compared with age-matched men (21.7% vs. 11.9%, respectively) [10]. Moreover, sex-related differences in immune cell composition within the synovial fluid, such as a higher abundance of macrophages in women with knee OA, have been reported [11]. In our previous study, plasma progesterone concentration was associated with higher reported pain intensity in postmenopausal women during the decade following menopause [12]. However, the mechanism by which progesterone influences pain and inflammation in the synovial joint remains unexplored.

The present study therefore aims to define the contribution of progesterone to synovial inflammation in both postmenopausal women and men aged 49 years and above living with knee OA.

## 2. Results

### 2.1. Synovial Tissue from Women with Knee OA Older than 49 Years Exhibited Increased Abundance of Proteins Involved in the Protein Synthesis Machinery Compared to Age-Matched Men

Sex differences in synovial tissue from women and men (n = 9–10 per group) were investigated using proteomic analyses. Patient characteristics are presented in Table 1 and Supplementary Tables S1–S5. Statistical testing of the patient metadata revealed no statistically significant differences between the experimental groups for any of the clinical parameters analyzed.

**Figure 1:**
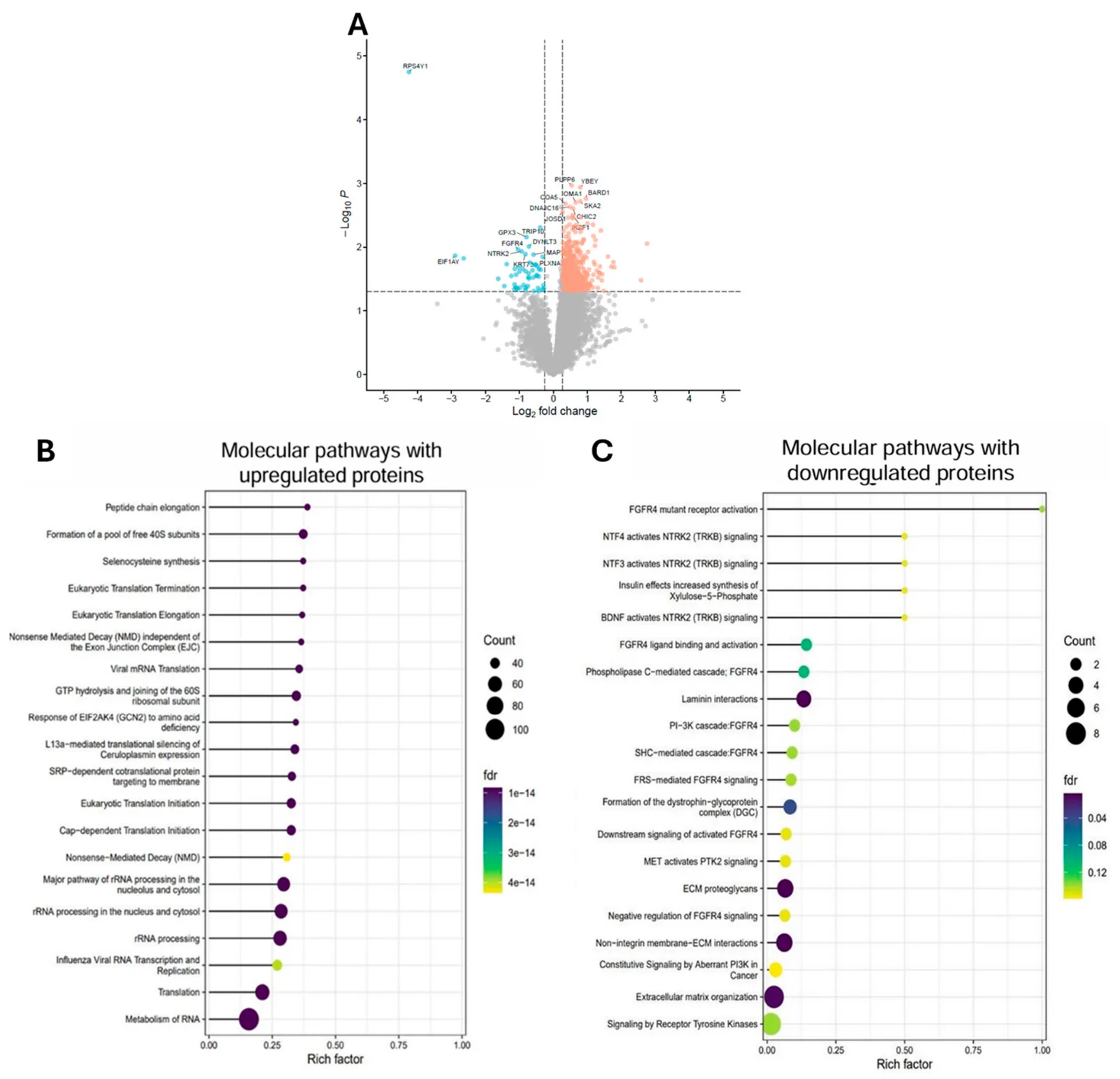
Proteins associated with the protein synthesis pathways were more abundant in women than men with knee OA. The synovial tissues of women with knee OA exhibit a protein expression profile characterized by higher abundance of proteins associated with translational and RNA metabolic processes and lower abundance of proteins linked to extracellular matrix organization, FGFR4 signaling, and neurotrophin pathways. (**A**) Volcano plot showing differential protein expression between women and men. Significantly upregulated proteins (pink; *p* < 0.05 and fold change ≥ 1.2) and significantly downregulated proteins (blue; *p* < 0.05 and fold change ≤ 0.8) in women are presented. Proteins that did not differ between sexes are shown in grey. The top 20 relevant and significantly enriched molecular pathways with (**B**) upregulated and (**C**) downregulated proteins in the synovial tissue of women with knee OA compared to men are shown.

A total of 8175 proteins were identified and quantified, of which 627 were significantly altered among the women compared to the men (*p* ≤ 0.05 and a log2-fold change ≥ 20%, Figure 1A). Pathway mapping showed that synovial tissues from women exhibit an upregulation of proteins regulating translational processes, including translation initiation, elongation, termination, and rRNA processing, together with increased activity of proteins involved in RNA metabolism pathways, including the RNA quality control pathways of nonsense-mediated RNA decay (Figure 1B).

Conversely, proteins associated with extracellular matrix (ECM) organization and adhesion, the activation of the fibroblast growth factor 4 receptor (FGFR4), and neurotrophic receptor tyrosine kinase (NTRK2) signaling were downregulated in the synovial tissues of women with knee OA compared to men (Figure 1C).

Among the upregulated pathways identified in the pathway enrichment analysis, the GCN2 (EIF2AK4)–amino acid deficiency stress-response pathway appeared alongside pathways related to translation initiation and elongation (Figure 1B). Although this combination may initially appear counterintuitive, as activation of GCN2 is classically associated with inhibition of global translation initiation through phosphorylation of eIF2α [13], closer examination of the proteins contributing to this pathway annotation indicated that it did not reflect activation of the GCN2 signaling pathway. EIF2AK4 (GCN2) itself was not detected in the proteomic dataset. Rather, the proteins assigned to the GCN2 pathway were predominantly ribosomal proteins (Supplementary Table S1).

### 2.2. Subjects with Similar Plasma Progesterone Levels Exhibited Similar Proteomic Profiles, Regardless of Sex

**Figure 2:**
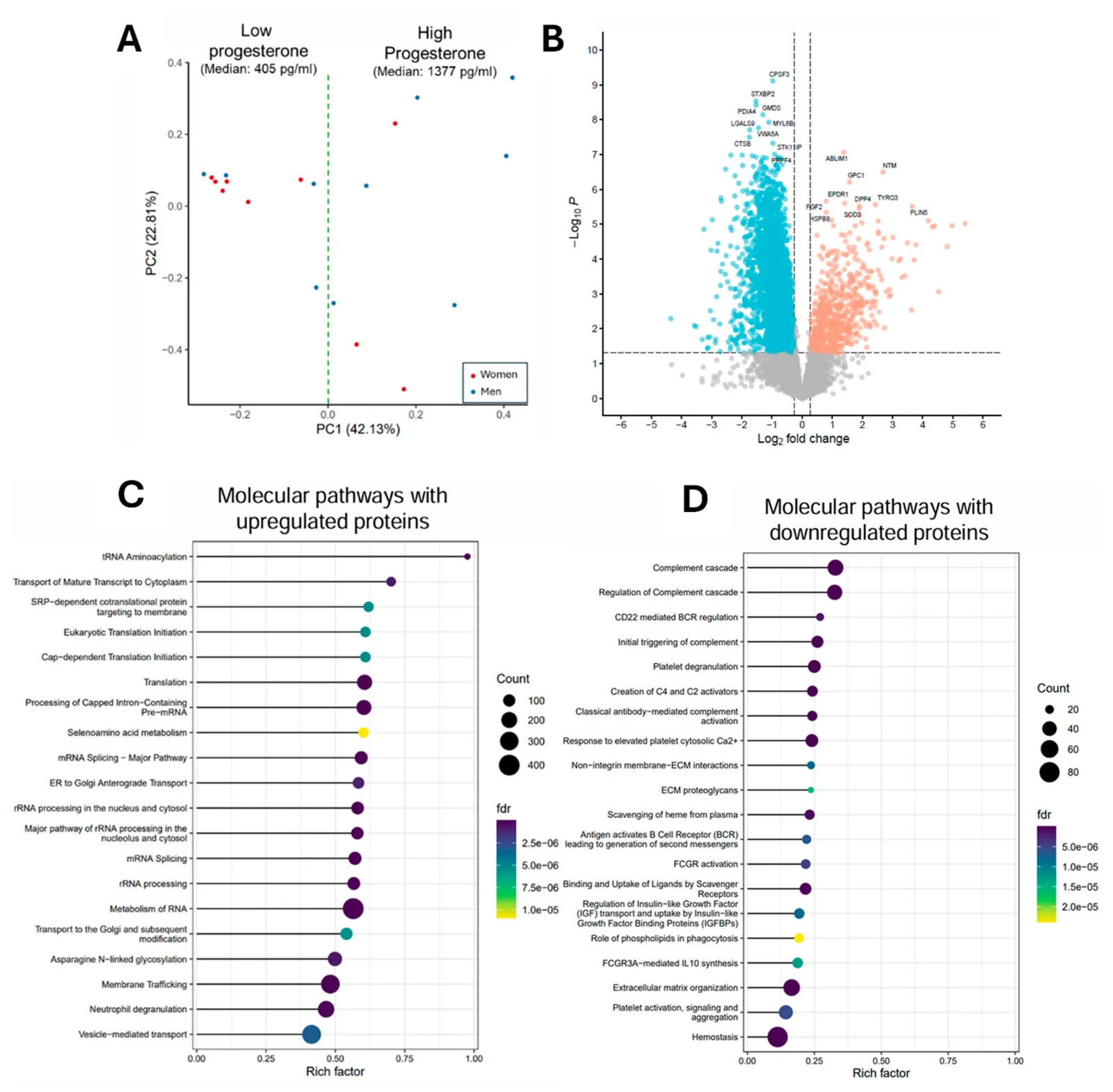
Low plasma progesterone levels are associated with increased activation of transcriptional and translational protein synthesis pathways and reduced activation of immune-related pathways in synovial tissue from OA patients. (**A**) Principal component analysis (PCA) of the proteomic profiles comparing synovial tissues collected from women versus men with knee OA, stratified by high versus low plasma progesterone concentrations. (**B**) Significantly upregulated proteins (pink; *p* < 0.05 and fold change ≥ 1.2) and significantly downregulated proteins (blue; *p* < 0.05 and fold change ≤ 0.8) in subjects with low and high plasma progesterone concentrations. Proteins whose expression did not change between groups are shown in gray. The top 20 relevant and significantly enriched molecular pathways with (**C**) upregulated and (**D**) downregulated proteins in the synovial tissue of subjects with low compared with high plasma progesterone concentrations are shown.

Principal component analysis (PCA) comparing the proteomes of synovial tissues from both sexes revealed the presence of two separate patient clusters regardless of sex: patients with low concentrations of progesterone (6 women and 4 men), with a median plasma concentration of 405 pg/mL (IQR 272–775 pg/mL), and patients with high concentrations of progesterone (3 women and 6 men), with a median plasma concentration of 1377 pg/mL (IQR 1020–1649 pg/mL) (Figure 2A). The median of all the samples together was 856 pg/mL (IQR 405–1406). No significant differences in age, BMI, radiographic score, or years from menopause onset were observed between the two groups (Supplementary Table S2). Notably, 1445 proteins were identified as differentially expressed between groups (*p* ≤ 0.05 and a log2-fold change ≥ 20%, Figure 2B). The following Reactome pathway analysis highlighted that subjects with lower plasma concentrations of progesterone also exhibited an upregulation in the expression of proteins involved in the translational processes and metabolism of RNA as well as the cellular transport of RNA (Figure 2C). Conversely, proteins involved in the activation and regulation of the complement cascade, platelet activation and hemostasis, B-cell receptor signaling, and activation of the FC gamma receptor (FcγR), as well as ECM interactions, were downregulated (Figure 2D).

To further explore whether the observed association between progesterone levels and the proteomic phenotype was independent of sex, we performed a linear regression analysis including progesterone concentration, sex, and their interaction. In this analysis, PC1 was positively associated with progesterone levels after accounting for sex (β = 0.051, *p* = 0.004), whereas sex itself was not significantly associated with PC1 after adjustment for progesterone (*p* = 0.941). Importantly, we found no evidence of a significant progesterone × sex interaction (β = 0.006, *p* = 0.865), suggesting that the relationship between progesterone levels and PC1 was similar in males and females in our cohort. Taken together, these findings suggest that the proteomic variation captured by PC1 may be more closely related to progesterone levels than to sex per se.

### 2.3. Elevated Global Poly(A)+ RNA Content Reflecting Increased Transcriptional Activity in OA Synovial Tissue from Women Was Localized to CD90^+^ Sublining FLS

**Figure 3:**
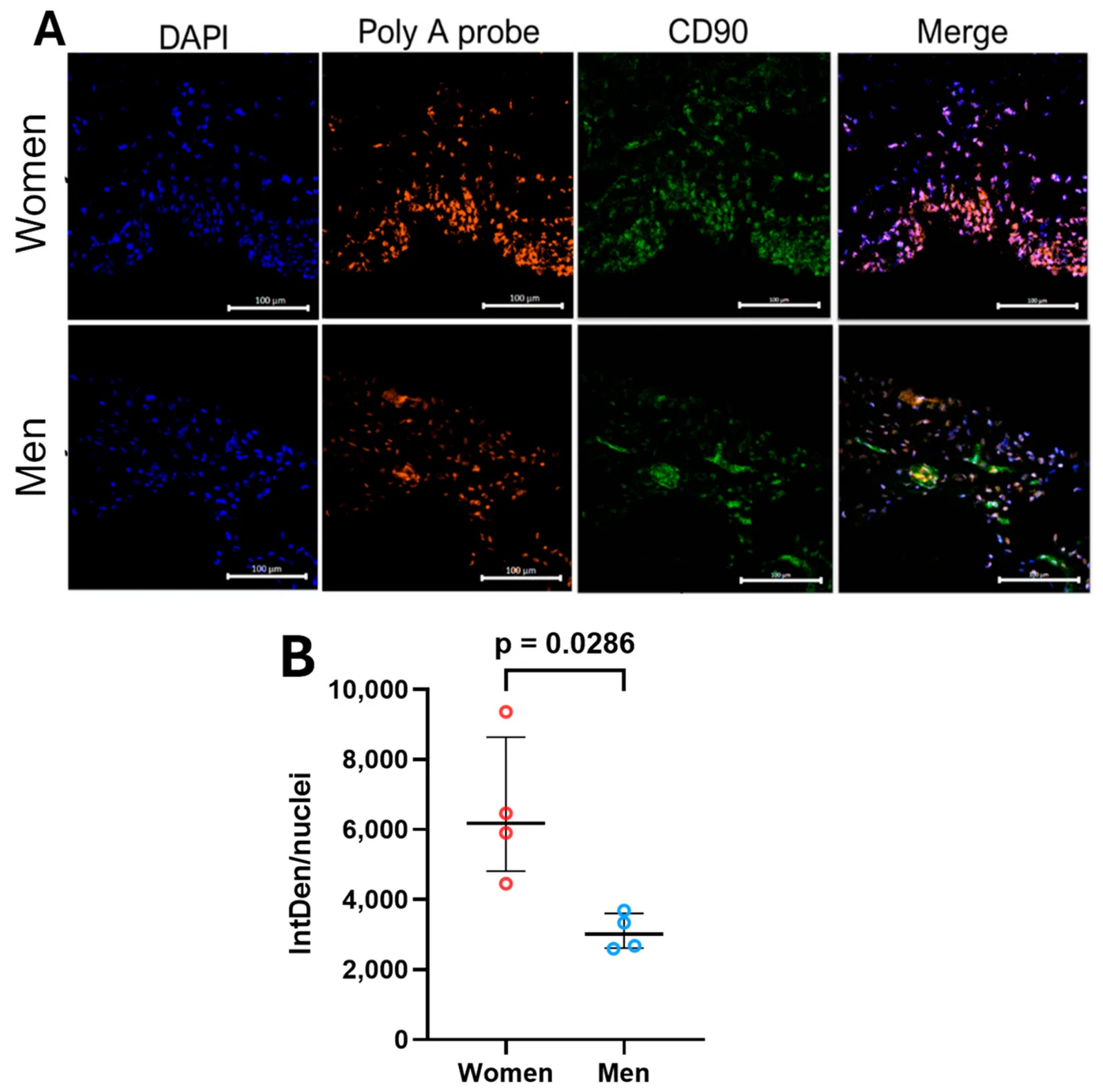
Increased RNA expression is localized to CD90^+^ FLS and is more pronounced in women compared to men. (**A**) Representative confocal images of the RNAscope in situ hybridization for total RNA in women and men. (**B**) Quantification of fluorescence intensity relative to total RNA (poly A probe) in synovial tissue, normalized by the number of DAPI-stained nuclei. Data are presented as median (IQR). Statistical significance was assessed using the Mann–Whitney U test. Scale bar: 100 µm.

RNAscope was used to further characterize the upregulated transcriptional profile in women identified by the initial proteomic analysis. For this purpose, a poly(A) probe was used in this RNAscope ISH to reflect overall transcriptional activity (Figure 3A). The quantification of the average signal intensity per cell (IntDen/nuclei) revealed a significantly higher signal intensity in synovial tissue from women compared to men (Figure 3B). The RNAscope was combined with immunofluorescence (IF) to quantify the differential distribution of mRNA across the synovial tissue and to elucidate which cell types were affected by the previously presented upregulation. The multiplex RNAscope ISH with CD90-IF labeling revealed that the increased RNA expression was highly localized to CD90^+^-FLS, also referred to as sublining FLS (Figure 3A,B; Supplementary Figure S1).

### 2.4. The Progesterone Receptor Is Expressed in CD90^+^ Sublining FLS and Does Not Show Any Sex Differences at the mRNA or Protein Level

**Figure 4:**
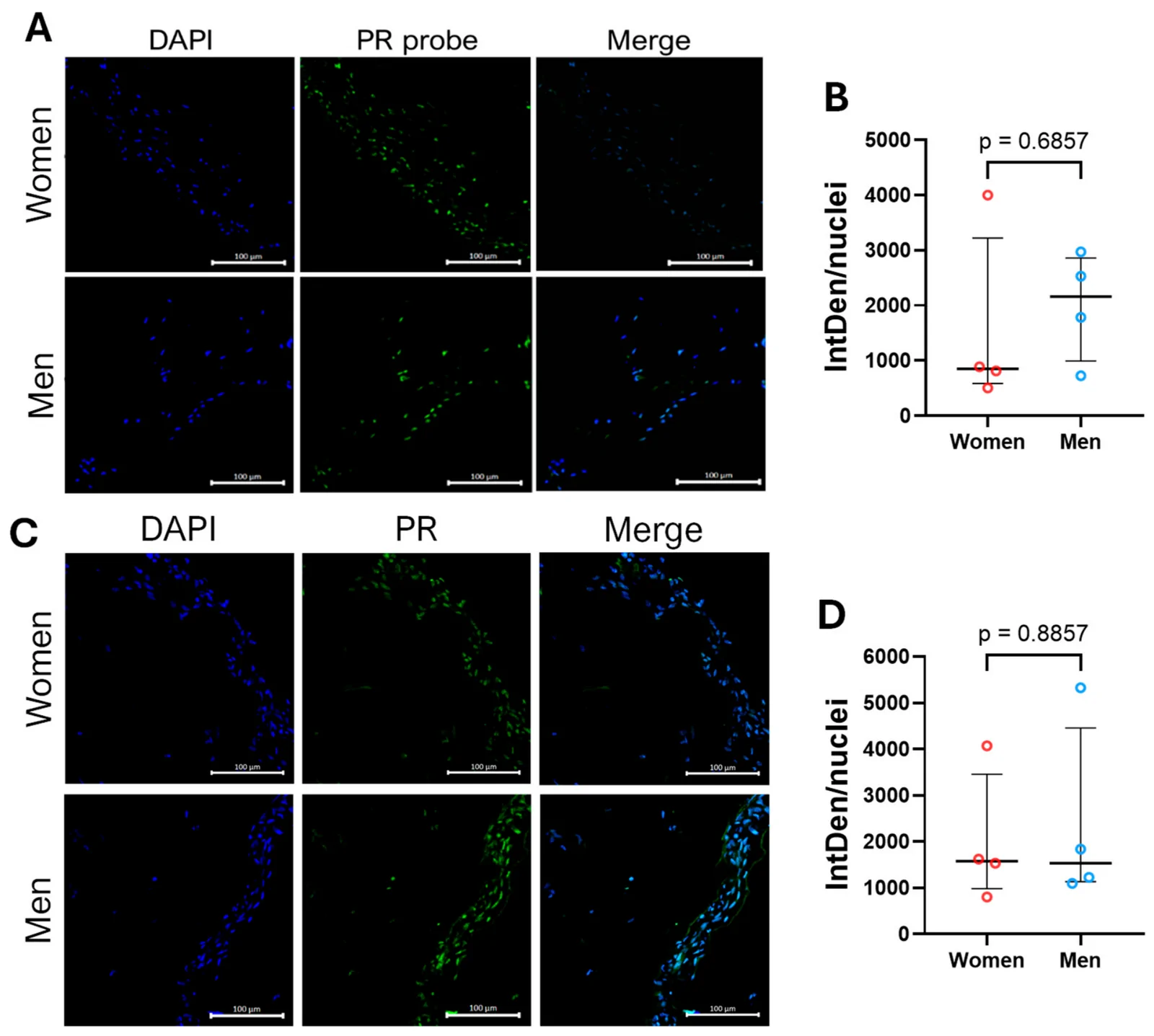
RNA and protein expression of the progesterone receptor did not differ between sexes. (**A**) Representative confocal images of the RNAscope in situ hybridization for progesterone receptor (PR) mRNA in the synovial tissue of women and men in the study. (**B**) Quantification of PR mRNA signal intensity normalized to DAPI-stained nuclei (fluorescence/DAPI). (**C**) Representative confocal images of immunofluorescence staining for PR protein (green) with DAPI nuclear counterstain (blue) in the synovial tissue of women and men. (**D**) Quantification of PR protein signal intensity normalized to DAPI-stained nuclei (fluorescence/DAPI). Data are presented as median (IQR). Statistical significance was assessed using the Mann–Whitney U test. Scale bar: 100 µm.

The multiplex RNAscope in situ hybridization (ISH) also included probes for progesterone receptor (PR) mRNA to further investigate the role of progesterone in FLS activity. The staining confirmed the presence of PR mRNA across the synovial membrane in both women and men and was predominantly co-localized with sublining FLS (Figure 4A; Supplementary Figure S2). Quantification of the average signal intensity per cell (IntDen/nuclei) demonstrated no significant difference in PR mRNA abundance between sexes (Figure 4B).

A separate IF staining for PR protein was performed following the RNAscope to validate the mRNA-level findings at the protein level. In line with the transcriptional findings, the results from this analysis revealed that synovial tissue exhibits detectable PR protein in both women and men in the study (Figure 4C; Supplementary Figure S3). The average signal intensity per cell (IntDen/nuclei) quantification for PR protein similarly showed no sex-specific differences among the subjects of the study (Figure 4D).

### 2.5. Isolated FLS from Women with Knee OA Present a Proteomic Signature Associated with Pro-Inflammatory and Pro-Fibrotic Processes

**Figure 5:**
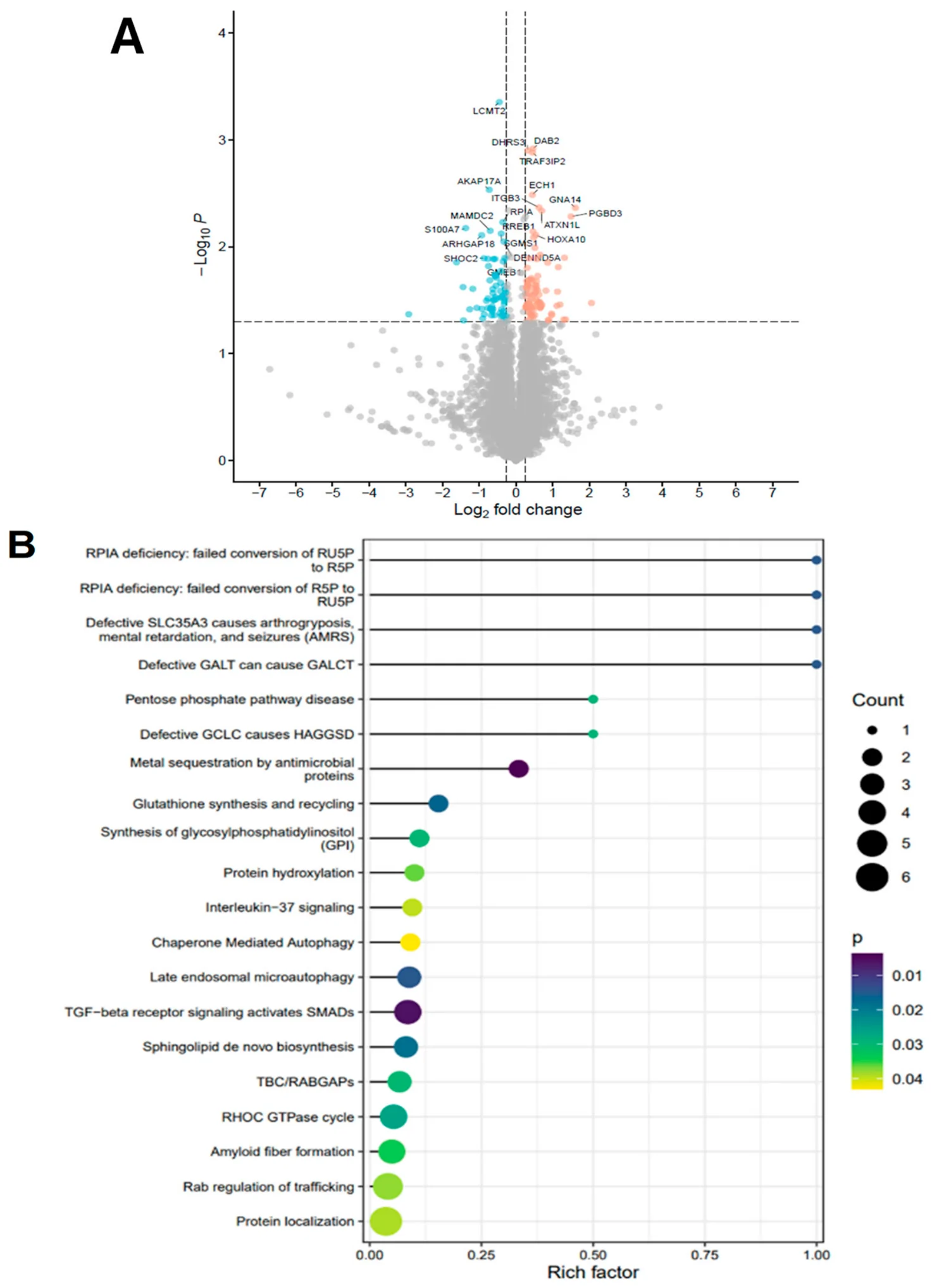
FLS isolated from women with knee OA showed enrichment of proteins associated with pro-fibrotic and pro-inflammatory pathways. (**A**) Volcano plot showing differential protein expression between women and men with knee OA. Significantly upregulated proteins (pink; *p* < 0.05 and fold change ≥ 1.2) and significantly downregulated proteins (blue; *p* < 0.05 and fold change ≤ 0.8) in women are presented. Proteins whose expression did not change between groups are shown in gray. (**B**) Top 20 relevant and significantly enriched biological pathways in FLS isolated from women with OA compared with men.

A proteomic analysis was also conducted on isolated FLS from the synovial tissue of women and men with knee OA. A total of 8175 proteins were identified across the proteomic dataset. The regulation analysis identified 183 proteins with significantly altered expression profiles in women compared to men (*p* ≤ 0.05 and log2-fold change ≥ 20%; Figure 5A). Pathway enrichment analysis identified several Reactome pathways with nominal *p*-values < 0.05 (Figure 5B). However, these associations did not remain significant after correction for multiple testing using the false discovery rate (FDR). The nominal *p*-values are presented to describe the observed enrichment patterns, while the corresponding FDR-adjusted values are provided in Supplementary Figure S4. The enrichment analyses revealed that FLS isolated from women exhibited several notable changes in protein expression of metabolic and immune pathways (Figure 5B), including reduced glycosaminoglycan production due to deficiencies in SLC35A3 and GALT [14,15], altered glucose metabolism associated with RPIA deficiency, and dysfunction in the catalytic subunit of glutamate cysteine ligase (GCLC), linked to early senescence. Additionally, these cells showed increased glutathione synthesis and activation of TGF-β/SMAD signaling, consistent with a proteomic signature associated with fibrotic processes [16,17,18]. The pathway enrichment analysis identified an “amyloid fiber formation” pathway among the pathways associated with the sex-related proteomic differences. However, closer examination of the proteins contributing to this annotation indicated that this pathway assignment was driven by automated database annotation rather than by proteins with established roles in amyloidogenesis. The proteins assigned to this pathway included Furin [16], Histone H4, Histone H2B [17], type 1-K [17], and Tetraspanin-15 [18], which are primarily involved in precursor protein processing, chromatin organization, and membrane protein regulation (Supplementary Table S2). Elevated levels of proteins involved in autophagy, vesicle transport, protein localization, and the anti-inflammatory protein IL-37 were also observed [19].

### 2.6. Progesterone Inhibits De Novo Protein Synthesis in FLS

**Figure 6:**
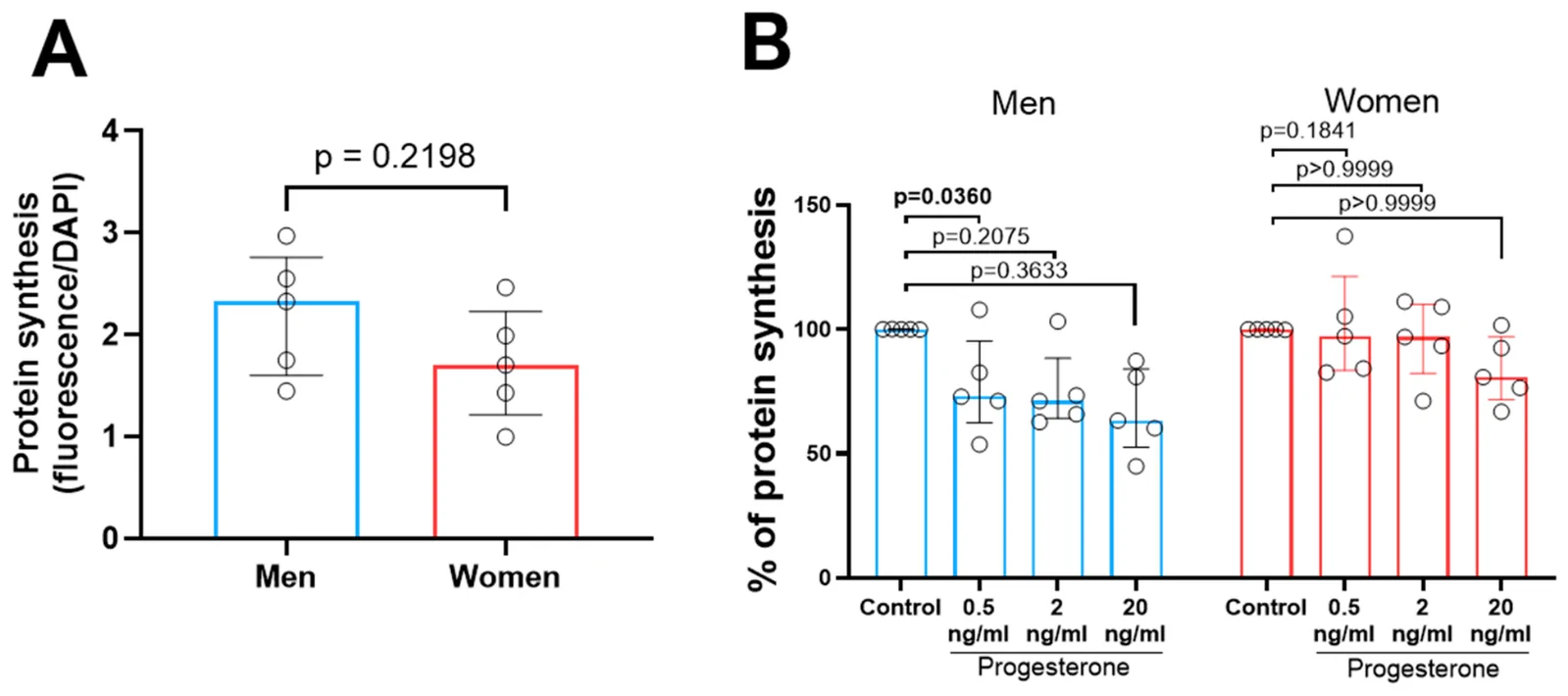
Physiological concentrations of progesterone inhibit *de novo* protein synthesis in cultured FLS isolated from men with knee OA. *De novo* protein synthesis was measured in FLS isolated from women (red) and men (blue) with knee OA after 4 h of incubation with progesterone (0.5, 2, and 20 ng/mL). (**A**) Quantification of *de novo* protein synthesis signal intensity normalized to DAPI-stained nuclei (fluorescence/DAPI). (**B**) *De novo* protein synthesis in men and women across three concentrations of progesterone treatment compared to baseline control (vehicle-treated cells). Data are presented as median (IQR). For comparisons among the dose groups, a Kruskal–Wallis test was performed, followed by Dunn’s post hoc test for pairwise comparisons between each dose group and the shared control.

Following the proteomic analysis and identification of PR protein and mRNA, the effects of FLS treatment with progesterone on *de novo* protein synthesis were assessed using FLS isolated from women and men with knee OA. Under basal conditions, without any treatment, the quantification results of the average signal intensity per cell indicate that *de novo* protein synthesis did not differ significantly between women and men in the study (Figure 6A). Instead, progesterone at 0.5 ng/mL reduced the amount of newly synthetized proteins only in men compared to baseline. A trend of reduction in protein synthesis was detected at higher concentrations, although not statistically significant (Figure 6B).

## 3. Discussion

Sex-related differences in OA, in terms of both incidence and prevalence, as well as severity and inflammatory responses, have been widely reported, particularly following menopause [20]. These differences are often attributed to the decline and dysregulation of gonadal hormone signaling. In the present study, we observed increased global RNA transcript levels, consistent with increased transcriptional activity, but also an upregulation of protein synthesis pathways in the synovial tissue from women compared to age-matched men with knee OA. These findings suggest enhanced cellular biosynthetic activity, potentially reflecting increased proliferation and activation of synovial cells, particularly FLS, in women. A previous study showed that activated FLS exhibit a marked increase in biosynthetic activity, involving both transcriptional and translational machinery [21]. In parallel, enhanced activity of the mTOR signaling pathway has been consistently observed in activated FLS, as evidenced by increased phosphorylation of downstream targets such as S6 kinase and 4E-BP1, as well as elevated levels of phosphorylated ribosomal protein S6, reflecting increased ribosomal activity and translation initiation [22,23,24,25].

Further stratification of patients based on circulating progesterone levels revealed that a low level of progesterone, independent of sex, differentiates individuals exhibiting a more pronounced inflammatory phenotype. This finding indicates that progesterone, or its downstream signaling pathways, could play a modulatory role in controlling the magnitude and persistence of inflammatory responses in OA, particularly within FLS. Indeed, the anti-inflammatory properties of progesterone are well established across multiple tissue types. In fibroblasts derived from the uterine cervix and various mucosal tissues, progesterone has been shown to suppress activation of nuclear factor kappa B, a central transcription factor driving inflammatory gene expression [26,27,28]. Through this mechanism, progesterone reduces the expression of pro-inflammatory mediators, including matrix metalloproteinases, which contribute to extracellular matrix degradation, and downregulates the production of chemokines responsible for immune cell recruitment [29]. In addition, progesterone plays a critical role in tissue repair and remodeling. During wound healing, it helps maintain a balanced repair response by limiting excessive production of transforming growth factor beta (TGF-β), thereby reducing the risk of pathological fibrosis [30,31,32].

Using RNAscope technology, we demonstrated increased global RNA transcript levels in synovial cells. These results provide spatial support for the localization and enrichment of RNA in CD90^+^ sublining FLS. We also provide the first evidence of progesterone receptor expression in CD90^+^ sublining FLS of the human synovium. Despite this, progesterone receptor expression at both the RNA and protein levels did not differ significantly between sexes, suggesting that the observed sex-specific differences are more likely driven by variations in circulating hormone levels or receptor signaling rather than receptor abundance alone.

The proteomic analysis of isolated FLS cultured under hormone-free conditions revealed that cells derived from women retained a more pro-fibrotic and pro-inflammatory signature compared to cells derived from men. This finding points to a degree of intrinsic cell priming that persists independently of the gonadal hormonal environment. However, under these in vitro conditions, no significant differences in global protein synthesis were observed between sexes. This may be explained by the absence of key modulatory factors, such as inflammatory stimuli or circulating hormones, which are present in vivo and are known to influence cellular activity. It may also be explained by factors such as proteome degradation, enzymatic digestion, culture selection, passaging, and the absence of crosstalk through, e.g., macrophage, endothelial, and immune signaling. Indeed, the hormonal milieu is a critical determinant of receptor signaling dynamics. For example, estrogens are known to upregulate progesterone receptor expression [33], while coregulatory proteins further modulate receptor activity and downstream transcriptional responses [34].

In line with this, our in vitro experiments showed that isolated OA FLS did not exhibit sex differences in basal protein synthesis. Furthermore, exposure to progesterone at concentrations corresponding to physiological circulating levels resulted in reduced protein synthesis in male-derived FLS, whereas no significant effect was observed in female-derived FLS. This differential responsiveness suggests that female FLS may require additional hormonal stimuli, such as those mediated through estradiol signaling, or may be more sensitive to inflammatory cues to fully engage progesterone-dependent pathways. Supporting this notion, previous studies have demonstrated that progesterone can exert enhanced biological effects in the presence of inflammatory mediators. In human cervical fibroblasts, IL-1β increases the expression of AKR1C1 and AKR1C2 and promotes progesterone metabolism to less active metabolites via these enzymes, suggesting enhanced local progesterone catabolism under inflammatory conditions [35]. Progesterone and IL-1β have also been shown to synergistically enhance activin A expression in cultured human endometrial stromal cells, indicating that IL-1β can augment progesterone-mediated cellular responses in uterine stromal contexts [36]. Therefore, the FLS proteomic findings may reflect a combination of in vivo priming and culture-selected features rather than capturing the complex native in vivo phenotype of FLS. A future approach to more closely preserve the native synovial microenvironment could be the use of synovial tissue explants, which have been previously utilized and even co-cultured with cartilage [37]. Moreover, the non-significant effects at the higher doses of progesterone in FLS isolated from male participants may reflect insufficient power rather than a true absence of biological effect.

Our findings suggest several questions regarding the sources and potential effects of progesterone in OA. Although BMI was available in our study, it does not provide information on body fat distribution or composition. This is particularly relevant given that adipose tissue may influence both inflammatory processes and the hormonal milieu, potentially affecting progesterone levels and activity [38,39]. Moreover, macrophages, which play an important role in OA pathogenesis and are responsive to progesterone, may represent an additional link between progesterone signaling, inflammation, and disease progression [40]. Further studies will therefore be important to clarify the mechanisms underlying the associations observed in our study. In particular, the inclusion of male and female non-OA control samples would help determine whether the observed differences reflect sex-related characteristics, disease-associated changes, or a combination of both.

**Figure 7:**
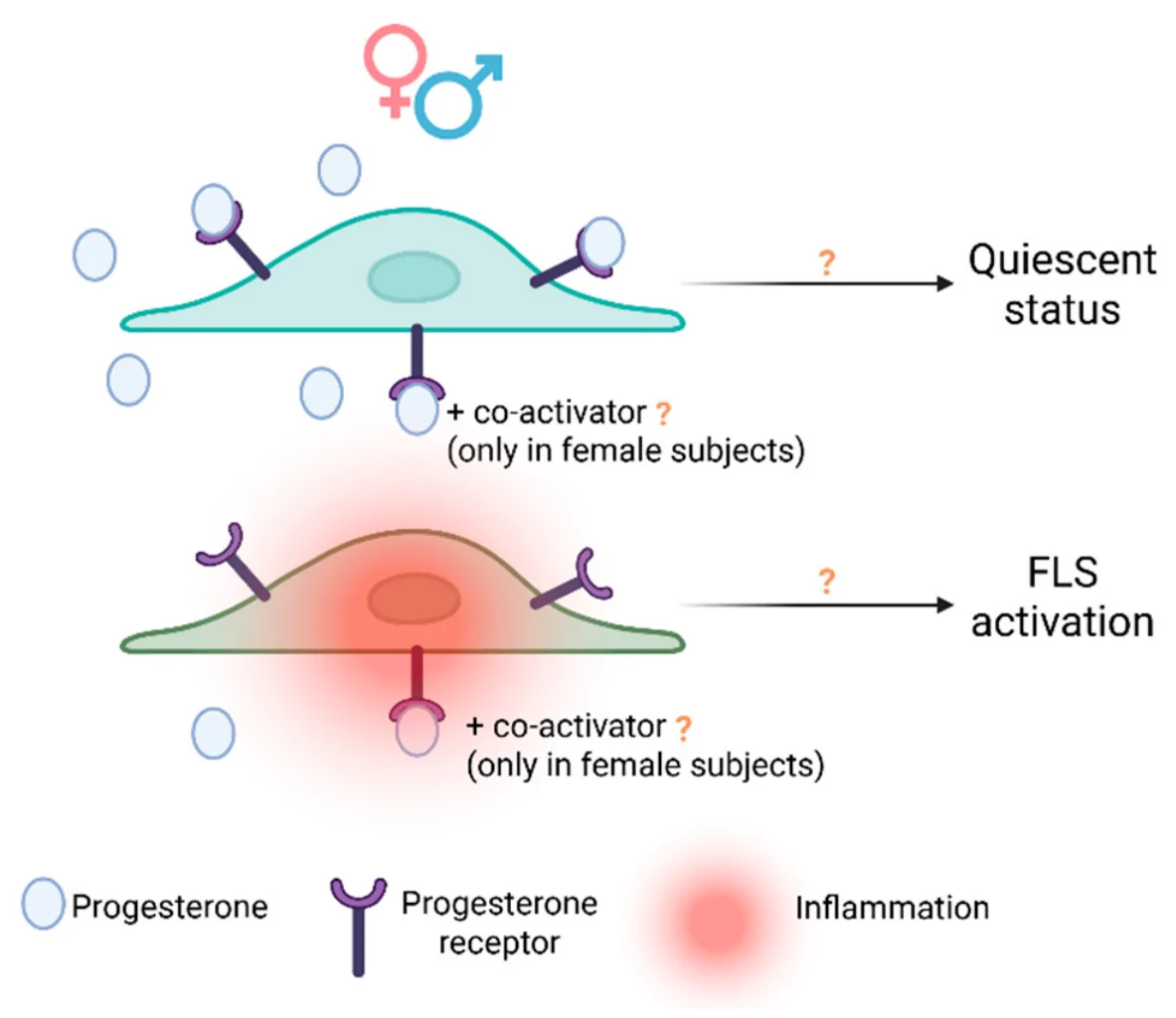
Conceptual model illustrating the potential role of progesterone in regulating CD90^+^ sublining FLS in knee OA. Under healthy conditions (**top**), physiological progesterone levels may contribute to maintaining CD90^+^ FLS in a relatively quiescent state and supporting synovial homeostasis. During inflammation (**bottom**), FLSs become activated and may promote the release of catabolic and pro-inflammatory mediators. Progesterone may modulate FLS activation and limit the production of pro-inflammatory and catabolic mediators. Under conditions of lower progesterone availability, such as after menopause, this potential regulatory effect may be reduced, which could contribute to increased FLS activation and sustained synovial inflammation. This mechanism is independent of sex, as both men and women express progesterone receptors at comparable levels in CD90^+^ FLS. However, in females, progesterone receptor activity may require a co-activator (e.g., estradiol) for full transcriptional function.

In summary, these findings suggest that progesterone may act as a physiological “brake” on the inflammatory activity of synovial fibroblasts (Figure 7). By attenuating pro-inflammatory signaling pathways and reducing the secretion of degradative and chemotactic factors, progesterone likely promotes a shift in fibroblast phenotype from an activated, pro-inflammatory state toward a more quiescent and homeostatic profile. This transition may be critical in limiting chronic inflammation and tissue damage in OA.

This study has some limitations that should be considered. The relatively small sample size limits the statistical power. Furthermore, the absence of data from premenopausal women makes it difficult to fully dissect the role of hormonal fluctuations and the broader endocrine environment in OA pathophysiology. While comparisons with age-matched men provide valuable insights, a more informative approach would include direct comparisons between pre- and postmenopausal women. In conclusion, our findings highlight a complex, context-dependent role for progesterone in modulating synovial inflammation and fibroblast activity in OA. Sex differences in disease phenotype appear to be driven primarily by circulating hormone levels rather than receptor expression, with cells derived from women exhibiting a more activated and pro-fibrotic profile. These results underscore the importance of considering hormonal status in OA research and may have implications for the development of sex-specific diagnostic approaches and the advancement of sex-specific therapeutic strategies.

## 4. Methods

### 4.1. Study Participants

Study participants were recruited at the SportsMed Orthopedic Clinic, Carlanderska Hospital (Gothenburg, Sweden), from patients scheduled to undergo total knee replacement surgery. The study was approved by the Regional Ethics Committee of Gothenburg (approval number 1087-16).

Inclusion criteria were a diagnosis of knee OA, age ≥ 49 years, and a radiographic severity grade ≥ 2 according to the Ahlbäck classification [41]. Exclusion criteria included a reported history of rheumatic disease, liver or kidney disease, cancer, or chronic pain disorders. Additionally, patients receiving opioid therapy or under medical treatment for mood or sleep disorders were not included.

All participants received written information about the study and provided informed consent. Initial written or verbal consent was obtained at the time of recruitment. On the day of surgery, written informed consent was confirmed, background information was collected, and preoperative blood samples were obtained.

Demographic and clinical background data were collected using a self-administered questionnaire, including age, height, weight, use of medications for OA-related symptoms, and other comorbidities under medical treatment. Female participants completed an additional questionnaire section covering age at menarche, age at menopause, number of pregnancies, and use of hormone replacement therapy and/or hormonal contraceptives. At the time of sampling, none of the female participants were using hormone replacement therapy or hormonal contraception. Three participants reported previous use of combined oral contraceptives; however, all had discontinued their use prior to study inclusion.

All surgical procedures were performed between 08:00 and 16:00, with preoperative blood collected between 7:30 and 13.00. The clinical characteristics of the study participants are summarized in Table 1 and Supplementary Tables S3–S7.

### 4.2. Plasma Collection

Prior to surgery, whole blood was collected by venipuncture into BD Vacutainer K2EDTA Plus blood collection tubes (Becton, Dickinson and Co., Allschwil, Switzerland). Plasma was separated by centrifugation at 1000× *g* for 10 min at 4 °C, transferred to sterile tubes, and stored at −80 °C until analysis.

### 4.3. Measurement of Sex Steroid and Sex Hormone-Binding Globulin Concentration in Plasma

Plasma concentration of progesterone was measured using commercially available Enzyme-Linked Immunosorbent Assay (ELISA) Kits (ThermoFisher Scientific, Waltham, MA, USA) as per the manufacturer’s instructions. The absorbance was read using the SpectraMax i3x Multi-Mode Microplate Reader (Molecular Devices, San Jose, CA, USA) and SoftMax Pro software (Version 6.5.1, Molecular Devices, San Jose, CA, USA).

### 4.4. Collection and Processing of Synovial Tissue

The synovial tissue was collected during total knee replacement surgery. The tissue was excised and immediately stored in cold HyClone Dulbecco’s Modified Eagle Medium (DMEM) with high glucose (Cytiva, Logan, UT, USA), 1% Penicillin–Streptomycin, 0.1% Amphotericin B, and 0.1% Gentamycin (Gibco, Grand Island, NY, USA) until the next step (approximately 2 h from the collection). The synovial tissue samples were then washed in Dulbecco’s phosphate-buffered saline (DPBS) (Cytiva, Logan, UT, USA) and divided into 3 × 3 mm sections, which were immediately flash-frozen in liquid nitrogen and then stored at −80 °C until the proteomic analysis. Other portions of the synovial tissue were stored in Optimal Cutting Temperature compound before freezing (OCT; ThermoFisher Scientific, Waltham, MA, USA) for histological and RNAscope evaluation or processed for the isolation of the fibroblast-like synoviocytes (FLSs).

### 4.5. Isolation of Fibroblast-like Synoviocytes

The harvested tissue was divided into 1 × 1 mm sections and added to DMEM containing 1% P/S, 0.1% Amphotericin B, and 0.1% Gentamycin. The sections were then centrifuged at 232× *g* for 10 min, after which the medium was removed. The tissue pellet was digested at 37 °C for 1 h with gentle shaking through the addition of 10 mL of pre-warmed digestion solution comprising DMEM, 1% P/S, 100 µg/mL Liberase TL (Roche Diagnostics GmbH, Mannheim, Germany), and 100 µg/mL DNase I (Roche Diagnostics GmbH, Mannheim, Germany). The digested sample was rinsed with DMEM containing 1% P/S, 0.1% Amphotericin B, 0.1% Gentamycin, and 10% FetalClone III Serum (Cytiva, Logan, UT, USA; also referred to as “complete medium”) and passed through a 40 µm filter to isolate the FLS. The cell suspension was centrifuged at 232× *g* for 8 min, and the supernatant was discarded. Finally, the cell pellet was resuspended in complete medium and cultured in a T75 flask (Sarstedt AG & Co. KG, Nümbrecht, Germany). The cells were maintained for 3 passages with regular changes in DMEM, 1% P/S, and 10% FetalClone III Serum, after which they were plated for proteomic analysis or used in the *de novo* protein synthesis assay.

### 4.6. Proteomic Analysis

Preparation of FLS. Isolated FLS were seeded on a 6-well plate (200,000 cells/well). After 16 h, the cells were washed and starved using only DMEM and 1% P/S for 2 h, after which they were incubated with DMEM supplemented with 1% P/S and charcoal-stripped FBS (Gibco, NY, USA) for 4 h. Then, the medium was removed, and the cells were washed in DPBS and dissociated with TrypLE Express (Gibco, NY, USA). The suspended FLS were centrifuged at 144× *g* for 10 min, and the resulting cell pellet was stored at −80 °C until the proteomic analysis.

Experimental analysis. Details for the experimental analysis are provided in Supplementary Information S1. Relative quantification was performed to compare protein expression in the knee OA synovial tissue in FLS isolated from the synovial tissue of 5 women and 5 men with knee OA.

During sample processing, one female sample was inadvertently analyzed twice, while a second female sample was not included in the analysis. Consequently, the progesterone-stratified proteomic analysis comprised 19 unique subjects: 10 male and 9 female subjects. The duplicated sample was treated as a single subject in the analysis, and the female sample that was not analyzed was excluded.

Proteins were extracted in SDS buffer, and protein concentrations were determined using the Bicinchoninic acid assay (BCA) [42] Samples were processed using a modified SP3 method. Briefly, 30 μg aliquots of the samples were reduced using 100 mM DTT and alkylated with 20 mM iodoacetamide. Proteins were precipitated on the Sera-Mag™ SpeedBeads (Cytiva) with acetonitrile, washed, and dried at room temperature. Beads were resuspended in 100 mM HEPES, and the proteins were digested with Trypsin/Lys-C mix [1:25] for two hours and trypsin [1:50] overnight. Beads were removed, and peptides were labeled using TMTpro 16-plex isobaric mass tagging reagents (Thermo Fisher Scientific, Waltham, MA, USA) according to the manufacturer’s instructions. The labeled samples were pooled into two Tandem Mass Tag (TMT) sets and purified by HiPPR Detergent Removal Resin and Pierce™ Peptide Desalting Spin Columns (both Thermo Fisher Scientific, Waltham, MD, USA). The TMT set was fractionated into 36 fractions using basic reversed-phase chromatography (bRP-LC, pH10) over 70 min. Each fraction was analyzed on an Orbitrap Eclipse™ Tribrid™ mass spectrometer equipped with the FAIMS Pro ion mobility system interfaced with the nLC 1200 liquid chromatography system (all Thermo Fisher Scientific, Waltham, MA, USA). Peptides were separated on a C18 35 cm column over 90 min, and data were acquired with the SPS MS3 method.

### 4.7. De Novo Protein Synthesis Assay

Isolated FLSs from 5 women and 5 men with knee OA were seeded in a 96-well plate (10,000 cells/well) in DMEM with 1% P/S and 10% FetalClone III Serum (Cytiva, Logan, UT, USA). After 16 h, culture medium was removed and replaced with DMEM supplemented with charcoal-stripped FBS (Gibco, Grand Island, NY, USA) containing progesterone (Merck Life Science AB, Solna, Sweden) at three different concentrations: 0.5, 2, and 20 ng/mL. The medium was replaced after 4 h with a methionine-free DMEM (containing D-glucose, without L-glutamine, sodium pyruvate, L-methionine, and L-cystine; Gibco, ThermoFisher Scientific, Invitrogen, Frederick, MD, USA) supplemented with 200 µM of L-cystine and 4 mM of L-glutamine (ThermoFisher Scientific, Invitrogen, Frederick, MD, USA) and the component A (Homopropargylglycine) from the Protein synthesis kit (Click-IT™ HPG Alexa Fluor 488, ThermoFisher Scientific, Invitrogen, Frederick, MD, USA) according to the manufacturer’s instructions. After 30 min, the cells were washed with DPBS and fixed with 3.7% paraformaldehyde for 15 min. Cells were then permeabilized by using ice-cold methanol for 10 min. Newly synthesized proteins were detected using the Click-iT HPG kit, following the manufacturer’s instructions.

### 4.8. RNAscope In Situ Hybridization

A multiplex RNAscope in situ hybridization (ISH) was performed to detect total RNA content and progesterone receptor (PR) mRNA in synovial tissue samples, using a commercial RNAscope kit (Bio-Techne, Dublin, Ireland) according to the manufacturer’s instructions.

Synovial membrane tissue blocks embedded in OCT compound were sectioned at 15 μm thickness using a cryostat (Leica) and mounted onto Superfrost Plus slides (Thermo Fisher Scientific, Waltham, MA, USA). Slides were washed in PBS for 5 min to remove OCT, incubated at 60 °C for 30 min, and then dehydrated through graded ethanol (50%, 70%, and twice in 100%) for 5 min each at room temperature. Slides were air-dried for 5 min at room temperature before applying three drops of RNAscope Hydrogen Peroxide, followed by incubation for 10 min at room temperature. The reagent was removed by gently tapping the slides on absorbent paper, and slides were washed twice in distilled water.

For target retrieval, slides were placed in preheated distilled water at 99 °C for 10 s, then transferred to target retrieval solution (99 °C) for 5 min, followed by rinsing in distilled water and immersion in 100% ethanol for 3 min. Slides were dried in an incubator at 60 °C for 5 min. Three drops of RNAscope Protease III were applied to each tissue section, and slides were placed in an EZ-Batch slide holder and incubated at 40 °C for 30 min. Following protease treatment, slides were washed twice in distilled water.

Two drops of the target probe were applied to each section, and hybridization was carried out at 40 °C for 2 h. Slides were washed twice in wash buffer for 2 min each. Signal amplification was performed sequentially using AMP1 (30 min at 40 °C), AMP2 (15 min at 40 °C), and AMP3 (15 min at 40 °C), with two 2-min washes in wash buffer between each step.

Cell nuclei were counterstained with 4′,6-diamidino-2-phenylindole (DAPI) (provided in the RNAscope kit) for 30 s, and excess reagent was removed. Coverslips were mounted using ProLong Gold Antifade Mountant (Thermo Fisher Scientific, Waltham, MA, USA), and slides were stored protected from light at 4 °C until imaging with a confocal microscope.

### 4.9. Immunofluorescence Staining

Slides were washed in PBS for 5 min to remove OCT, incubated at 60 °C for 30 min, and then dehydrated through graded ethanol solutions (70%, 80%, 90%, and twice in 100%) for 5 min each at room temperature (RT). Tissue sections were subsequently incubated with Proteinase K (20 µg/mL; Sigma-Aldrich, Taufkirchen, Germany) at 37 °C for 30 min, followed by 10 min at RT. After washing in PBST for 5 min, the slides were blocked with 10% goat serum (MP Biomedicals, Irvine, CA, USA) in PBST for 30 min at RT.

The primary anti-PR antibody (1:166; Bio-Techne, Dublin, Ireland) was applied and incubated overnight at 4 °C. The following day, slides were washed three times in PBST and then incubated with the secondary antibody Alexa Fluor 647 (1:1500 dilution; Thermo Fisher Scientific, Waltham, MA, USA) for 30 min at RT. Excess secondary antibody was removed by washing three times in PBST. Coverslips were mounted using a mounting medium containing DAPI (Sigma-Aldrich, Taufkirchen, Germany), and slides were stored protected from light at 4 °C until imaging with a confocal microscope.

For samples undergoing both immunofluorescence staining and RNAscope, the primary antibody was applied after the amplification step of the RNAscope protocol. The primary antibody against CD90 (1:100; Thermo Fisher Scientific, Waltham, MA, USA) was added to the tissue and incubated overnight at 4 °C. Following three washes in PBST, slides were incubated with the secondary antibody Alexa Fluor 488 anti-sheep (Thermo Fisher Scientific, Waltham, MA, USA; 1:1500) for 30 min at RT.

Cell nuclei were counterstained with DAPI (provided in the RNAscope kit) for 30 s, and excess reagent was removed. Coverslips were mounted using ProLong Gold Antifade Mountant (Thermo Fisher Scientific, Waltham, MA, USA), and slides were stored protected from light at 4 °C until confocal imaging.

### 4.10. Data Analysis

Results from all ELISAs were analyzed by fitting the standard curves to a four-parameter logistic regression model, and sample concentrations were calculated using GainData^®^ software (Arigo Biolaboratories; https://www.arigobio.com/elisa-analysis). Fluorescence images were processed with ImageJ/Fiji software (ver. 1.54g) [43]. Total fluorescence intensity (densitometric signal) was normalized to the number of cells, determined by counting DAPI-positive nuclei. Statistical analyses were performed using GraphPad Prism (version 10.2.2; GraphPad Software, Inc., La Jolla, CA, USA).

Analysis of proteomic data. Raw files were processed and analyzed with Proteome Discoverer (ver 3.0, Thermo Fisher Scientific) by matching against UniProt Swiss-Prot human using Sequest as a search engine. Within the analysis workflow, the abundances of all proteins were normalized. Results for the synovial membrane and FLS were analyzed separately. Values of protein quantification data from both TMT sets were normalized by dividing the sample abundances by the reference abundances within each TMT set using the reference samples. Fold changes between sample groups were calculated, and then quantification data were log2-transformed for statistical testing using Welch’s *t*-test. Proteins with a fold change of >20% and a *p*-value < 0.05 were considered significantly altered between males and females.

The MS proteomics data have been deposited in the ProteomeXchange Consortium (http://proteomecentral.proteomexchange.org) via the PRIDE partner repository [44] with the dataset identifier PXD061120.

Significantly differentially expressed proteins were analyzed using Reactome pathway analysis (Reactome.org [45]) to identify overrepresented pathways. To identify the most relevant pathways affected in our biological system, pathways that are not relevant in the context of osteoarthritis were excluded. Statistical analyses regarding pathway enrichment were performed using R Version 4.4.1 (R Foundation for Statistical Computing, Vienna, Austria), and the R package ggplot2 3.51 was used for visualizations.

## Figures and Tables

**Table 1:** Characteristics of the study participants. No statistically significant differences were found between the experimental groups for any of the parameters analyzed (Mann–Whitney U test). Values are presented as median (interquartile range, IQR).

**Proteomic analysis of the synovial tissue: Women vs. Men**				
Sex	Age	BMI	Radiographic score	Pain intensity
Men (n = 10)	64 (59–69)	27.78 (25.99–30.25)	3 (3–4)	0.62 (0.35–0.77)
Women (n = 10)	61 (55–64)	30.47 (24.64–34.30)	3 (3–4)	0.64 (0.57–0.75)
**Proteomic analysis of the fibroblast-like synoviocytes (FLSs): Women vs. men**				
Sex	Age	BMI	Radiographic score	Pain intensity
Men (n = 5)	64 (61–74)	27.17 (24.17–30.38)	3 (3–4)	0.64 (0.49–0.87)
Women (n = 5)	61 (55–64)	30.48 (24.4–33.59)	3 (2.5–4)	0.65 (0.55–0.78)
***De novo* protein synthesis**				
Sex	Age	BMI	Radiographic score	Pain intensity
Men (n = 5)	63 (61–69)	27.73 (26.86–31.05)	4 (3–4)	0.68 (0.33–0.70)
Women (n = 5)	57 (56–63)	30.04 (25.87–33.11)	3 (2.5–3.5)	0.72 (0.31–0.80)
**RNAscope and immunofluorescence staining**				
Sex	Age	BMI	Radiographic score	Pain intensity
Men (n = 4)	57 (51–59)	30.95 (27.54–35.18)	3 (3–4)	0.64 (0.29–0.73)
Women (n = 4)	60 (59–61)	29.56 (24.2–36.25)	4 (3.25–4)	0.75 (0.55–0.79)

## Data Availability

The datasets used and/or analyzed during the current study are available from the corresponding author upon reasonable request. The mass spectrometry proteomics data have been deposited in the ProteomeXchange Consortium via the PRIDE [44] partner repository with the dataset identifier PXD061120.

## Supplementary Materials

The following supporting information can be downloaded at https://www.mdpi.com/article/10.3390/ijms27198899/s1.
