# Supplementary material for: Progesterone Modulates Protein Synthesis in Osteoarthritic Fibroblast-like Synoviocytes
Source: Int J Mol Sci. 2026 Oct 7;27(19):8899. doi: 10.3390/ijms27198899 (PMC13664916; doi:10.3390/ijms27198899)
Supplement: Supplementary file 1 [file ijms-27-08899-s001.zip › ijms-4580967-supplementary.pdf]

SUPPLEMENTARY INFORMATION

Supplementary information S1

Supplementary Table S1. **Proteins contributing to the automated GCN2 (EIF2AK4) pathway annotation.** The proteins assigned to the Reactome GCN2 (EIF2AK4) pathway in the pathway enrichment analysis are shown together with their UniProt identifiers, protein name and gene.

UniProt ID	Protein Name	Gene
**P23396**	Small ribosomal subunit protein uS3	*RPS3*
**P32969**	Large ribosomal subunit protein uL6	*RPL9*
**Q71UM5**	Ribosomal protein eS27-like	*RPS27L*
**P62917**	Large ribosomal subunit protein uL2	*RPL8*
**P18621**	Large ribosomal subunit protein uL22	*RPL17*
**P39019**	40S ribosomal protein S19	*RPS19*
**P42766**	60S ribosomal protein L35	*RPL35*
**P62277**	40S ribosomal protein S13	*RPS13*
**P62750**	60S ribosomal protein L23a	*RPL23A*
**P39023**	60S ribosomal protein L3	*RPL3*
**P63220**	40S ribosomal protein S21	*RPS21*
**P62910**	60S ribosomal protein L32	*RPL32*
**P62899**	60S ribosomal protein L31	*RPL31*
**P61247**	Small ribosomal subunit protein eS1	*RPS3A*
**P62851**	Small ribosomal subunit protein eS25	*RPS25*
**P08865**	40S ribosomal protein SA	*RPSA*
**P08708**	Small ribosomal subunit protein eS17	*RPS17*
**P26373**	Large ribosomal subunit protein eL13	*RPL13*
**P46781**	Small ribosomal subunit protein uS4	*RPS9*
**P62906**	Large ribosomal subunit protein uL1	*RPL10A*
**P36578**	60S ribosomal protein L4	*RPL4*
**P35268**	60S ribosomal protein L22	*RPL22*
**P42677**	Small ribosomal subunit protein eS27	*RPS27*
**P84098**	60S ribosomal protein L19	*RPL19*
**P15880**	Small ribosomal subunit protein uS5	*RPS2*
**Q02543**	60S ribosomal protein L18a	*RPL18A*
**P61254**	60S ribosomal protein L26	*RPL26*
**P62263**	40S ribosomal protein S14	*RPS14*
**P62241**	40S ribosomal protein S8	*RPS8*
**P62701**	40S ribosomal protein S4, X isoform	*RPS4X*
**P62888**	60S ribosomal protein L30	*RPL30*
**P62269**	40S ribosomal protein S18	*RPS18*
**P62841**	40S ribosomal protein S15	*RPS15*
**P62424**	60S ribosomal protein L7a	*RPL7A*
**P62249**	40S ribosomal protein S16	*RPS16*

Supplementary Table S2. **Proteins contributing to the automated "amyloid fiber formation" pathway annotation.** The proteins assigned to the "amyloid fiber formation" pathway in the Reactome enrichment analysis are shown together with their UniProt identifiers, gene and protein names.

UniProt ID	Protein Name	Gene
**P09958**	Furin	*FURIN*
**P62805**	Histone H4	*H4C1*
**O95858**	Tetraspanin-15	*TSPAN15*
**O60814**	Histone H2B type 1-K	*H2BC12*

Supplementary Table S3. **Characteristics of the study participants included in the proteomic analysis of the synovial tissue.**

ID/PCF ID/TMT Label	Sex	Age	BMI	Radiographic score	Pain intensity VAS (0-1)
1/T16/129N	M	69	24.11	4	0.64
2/T17/129C	M	57	36.11	4	0.19
5/T6/129N	M	64	28.38	3	0.40
10/T7/129C	M	70	29.03	3	0.07
17/T10/131N	M	67	29.54	3	0.81
19/T8/130N	M	59	32.37	3	0.75
23/T9/134N	M	79	27.17	3	0.58
31/T18/130N	M	63	24.22	4	0.98
36/T19/130C	M	57	26.88	3	0.59
63/T20/131N	M	64	26.58	3	0.64
**Median (IQR)**		**64**	**27.78**	**3**	**0.62**

		(59-69)	(25.99-30.25)	(3-4)	(0.35-0.77)
12/T1/126	F	64	30.48	3	0.65
15/T2/127N	F	55	35.43	4	0.84
16/T3/127C	F	57	34.29	4	0.34
21/T4/128N	F	61	24.8	4	0.50
30/T11/126	F	55	31.74	2	0.72
37/T12/127N	F	60	34.31	3	0.79
39/T13/127C	F	61	24.15	3	0.73
41/T14/128N	F	72	30.45	3	0.59
45/T5/128C	F	49	28.40	2	0.63
64/T15/128C	F	64	24	3	0.59
**Median (IQR)**		**61 (55-64)**	**30.47 (24.64-34.30)**	**3 (3-4)**	**0.64 (0.57-0.75)**

Supplementary Table S4. **Summary of the characteristics of the study participants included in the proteomic analysis of the synovial tissue comparing patients with low versus high concentration of progesterone. Values are presented as median (interquartile range, IQR).**

Parameter	Low progesterone	High progesterone
Progesterone concentration (pg/ml)	405 (272-775)	1377 (1020-1649)
Female sex (n/total)	6/10	3/9
Age (years)	61 (56-64)	64 (59-71)
BMI	27 (24-31)	30 (27.5-33)
Radiographic score	3 (3-4)	3 (3-4)
Years from menopause onset	8 (2.5-11)	10 (4-13)

Supplementary Table S5. **Characteristics of the study participants included in the proteomic analysis of the isolated FLS.**

ID/PCF ID/TMT Label	Sex	Age	BMI	Radiographic score	Pain intensity VAS (0-1)
1/C9/132C	M	69	24.11	4	0.64
5/C4/135N	M	64	28.38	3	0.40
19/C6/134N	M	59	32.37	3	0.75
23/C5/133C	M	79	27.17	3	0.58
31/C10/133N	M	63	24.22	4	0.98
**Median (IQR)**		**64 (61-74)**	**27.17 (24.17-30.38)**	**3 (3-4)**	**0.64 (0.49-0.87)**
12/C1/131C	F	64	30.48	3	0.65
15/C3/134C	F	55	35.43	4	0.84
21/C2/132N	F	61	24.8	4	0.50
30/C7/131C	F	55	31.74	2	0.72
64/C8/132N	F	64	24	3	0.59
**Median (IQR)**		**61 (55-64)**	**30.48 (24.4-33.59)**	**3 (2.5-4)**	**0.65 (0.55-0.78)**

Supplementary Table S6. **Characteristics of the study participants included in the *de novo* protein synthesis assay.**

ID	Sex	Age	BMI	Radiographic score	Pain intensity VAS (0-1)
3	M	61	27.73	4	0.68
11	M	60	33.69	4	0.69
32	M	69	27.17	4	0.27
36	M	68	26.54	3	0.70
54	M	63	28.41	3	0.38
**Median (IQR)**		**63 (61-69)**	**27.73 (26.86-31.05)**	**4 (3-4)**	**0.68 (0.33-0.70)**
8	F	57	27.64	3	0.85
27	F	60	24.09	4	0.30
30	F	55	31.74	2	0.72
52	F	65	34.48	3	0.32
53	F	56	30.04	3	0.75
**Median (IQR)**		**57 (56-63)**	**30.04 (25.87-33.11)**	**3 (2.5-3.5)**	**0.72 (0.31-0.80)**

Supplementary Table S7. **Characteristics of the study participants included in the RNAscope analysis and immunofluorescence staining.**

ID	Sex	Age	BMI	Radiographic score	Pain intensity VAS (0-1)
2	M	57	36.11	4	0.19
19	M	59	32.37	3	0.75
33	M	49	29.53	3	0.68
36	M	57	26.88	3	0.59
**Median (IQR)**		**57 (51-59)**	**30.95 (27.54-35.18)**	**3 (3-4)**	**0.64 (0.29-0.73)**
21	F	61	24.80	4	0.50
27	F	60	24.09	4	0.79
37	F	60	34.31	3	0.79
48	F	59	36.89	4	0.70
**Median (IQR)**		**60 (59-61)**	**29.56 (24.2-36.25)**	**4 (3.25-4)**	**0.75 (0.55-0.79)**

Supplementary Figure S1. **RNAscope analysis of total RNA expression in CD90 FLS.** Images from all individual patients included in the analysis are shown to illustrate the inter-individual variation within each sex group. The corresponding quantitative analysis is presented in Figure 3 B. Scale bar: 100 µm.

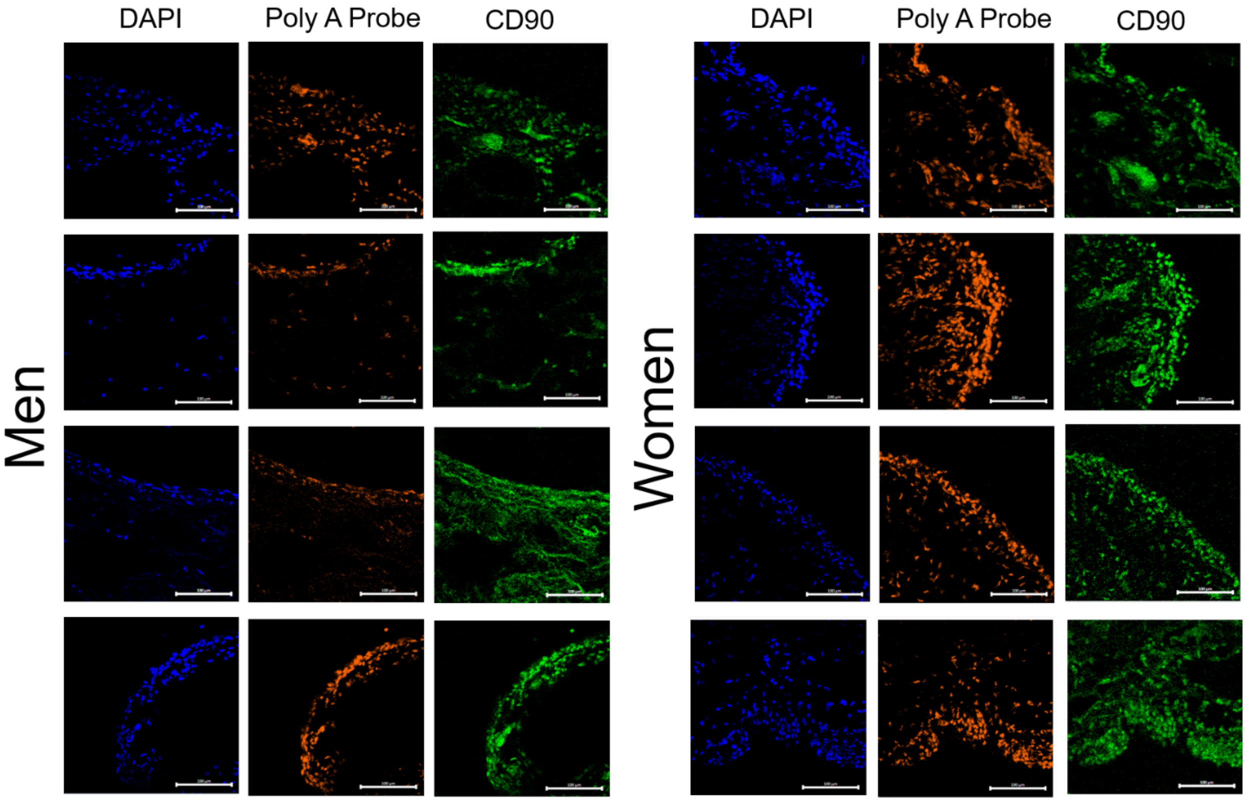

Supplementary Figure S2. **RNAscope analysis of PR expression in FLS.** Images from all individual patients included in the analysis are shown to illustrate the inter-individual variation within each sex group. The corresponding quantitative analysis is presented in Figure 4B. Scale bar: 100 μm.

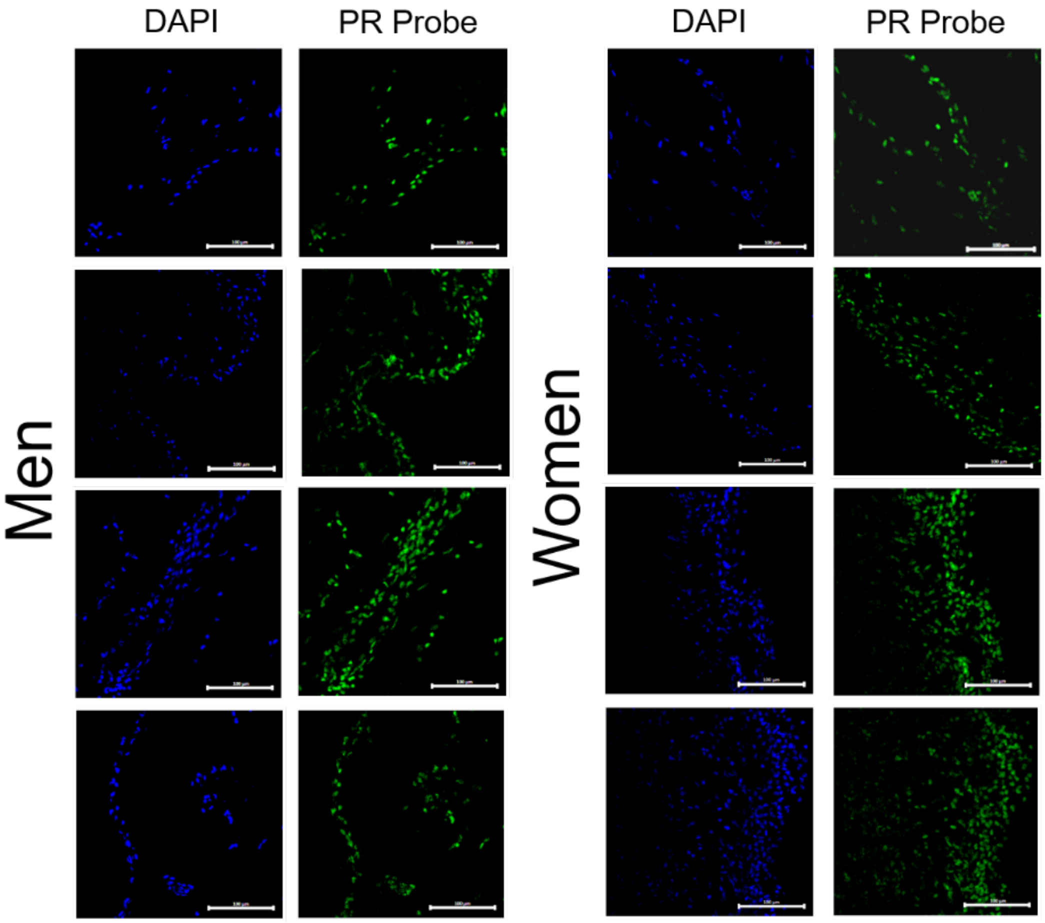

Supplementary Figure S3. **PR expression in FLS.** Images from all individual patients included in the analysis are shown to illustrate the inter-individual variation within each sex group. The corresponding quantitative analysis is presented in Figure 4D. Scale bar: 100 µm.

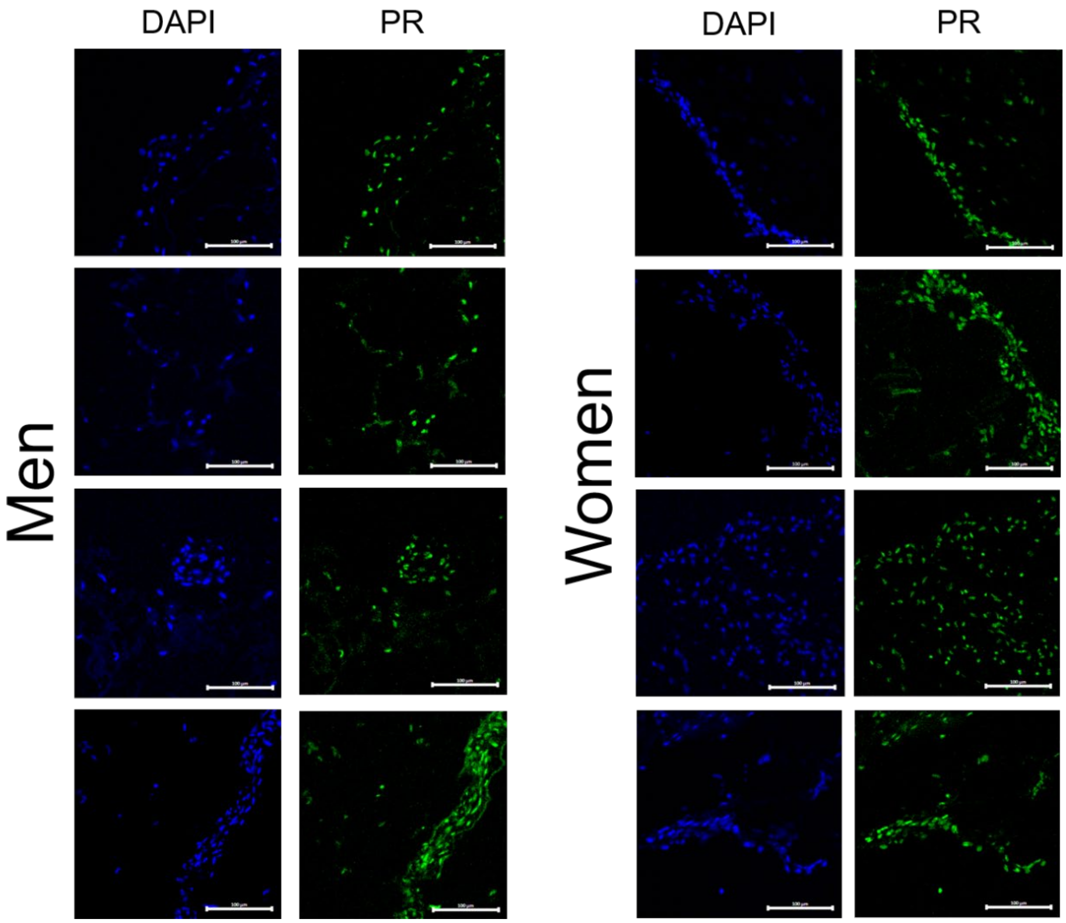

Supplementary Figure S4. **FDR-adjusted Reactome pathway enrichment analysis of sex-associated proteins.** Reactome pathway enrichment analysis was performed using proteins differentially expressed between FLS isolated from women and men with knee OA. The figure presents the FDR-adjusted P-values corresponding to the Reactome pathways shown in Fig. 5B.

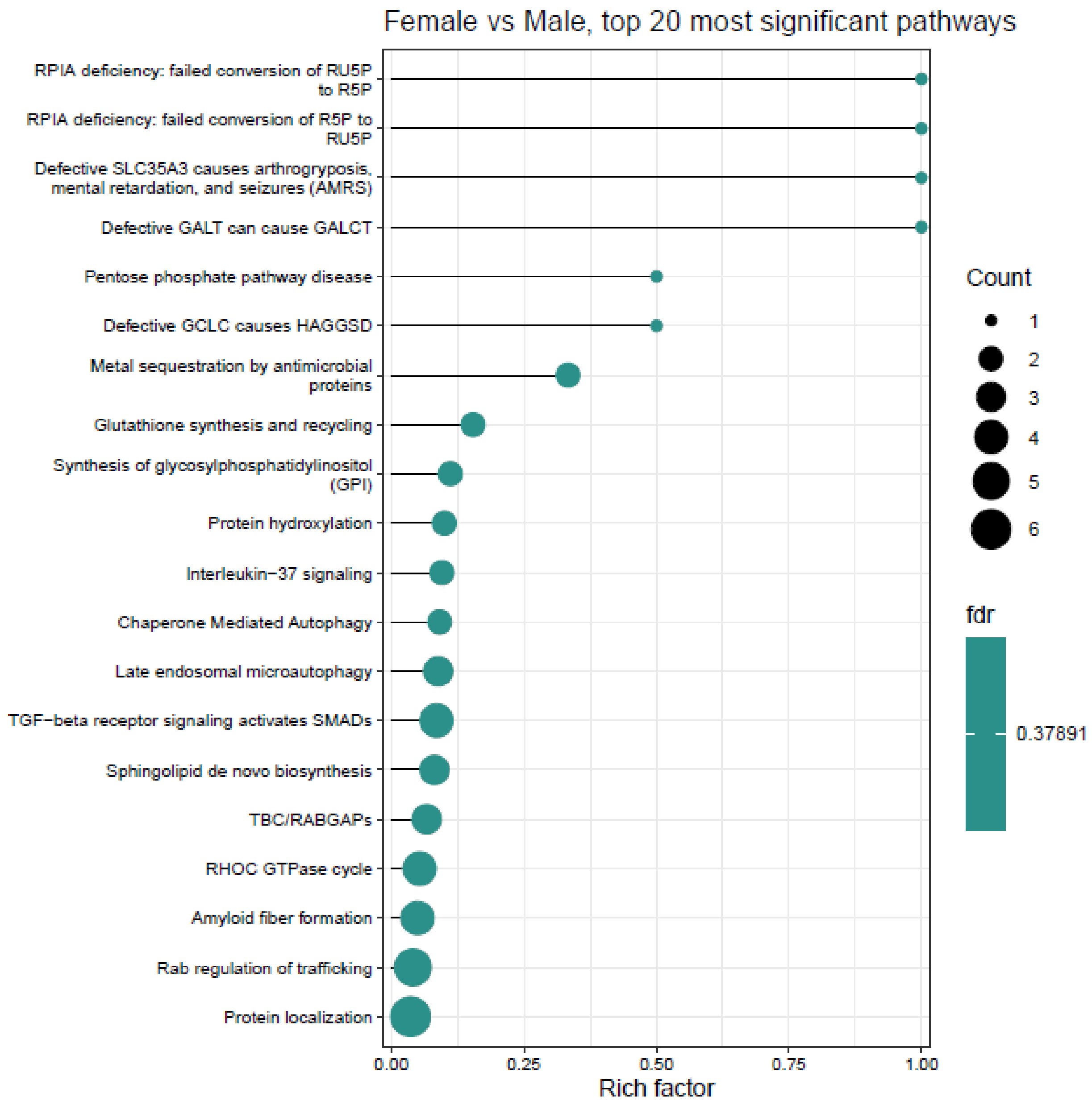

Supplementary information S2

Proteomic sample preparation

Relative quantification was performed to compare protein expression in synovial membrane and fibroblast-like synoviocytes collected from women and men with knee osteoarthritis.

The sample material was homogenized by bead-beating using a FastPrep-24 instrument (MP Biomedicals) in SDS buffer, and protein concentration was determined using Pierce BCA Protein Assay Kit (Thermo Scientific) on a SpectraMax iD3 (Molecular Devices).

Samples were processed using a modified SP3 method. Samples (30 µg) were reduced in 10mM dithiothreitol at 56°C for 30min and alkylated in 20mM iodoacetamide at room temperature for 30min. Washed hydrophobic and hydrophilic Sera-Mag™ SpeedBeads (Carboxylate-Modified, Cytiva) were added to the samples with a bead to protein ratio of 10:1. Proteins were precipitated on the beads by acetonitrile (final concentration 70%) , washed with 70% ethanol and dried at room temperature. Beads were resuspended in 50 µL of 50 mM HEPES and proteins were digested with Trypsin/Lys-C mix [1:25, Promega] for two hours and trypsin [1:50, Promega] overnight. The peptide supernatants were collected, the magnetic beads were washed with 50 µL of 50 mM HEPES, and the supernatants were combined with the initial peptide supernatant.

Samples were labelled using TMTpro 16-plex isobaric mass tagging reagents (Thermo Fisher Scientific). The labelled samples were pooled into two TMT sets, containing each tissue and cell samples, as well as sample C3 (PCF ID; isolated fibroblast from a woman with knee OA) and sample T3 (PCF, ID; synovial samples from a woman with knee OA), which served as references for normalization between the TMT sets.

Pooled peptide samples were purified using HiPPR Detergent Removal Resin and Pierce peptide desalting spin columns (both Thermo Scientific), according to the manufacturer´s instructions. The TMT sets were fractionated by basic reversed-phase chromatography on a Dionex Ultimate 3000

UPLC system (Thermo Fisher Scientific). Peptide separations were performed using a reversed-phase XBridge BEH C18 column (3.5 µm, 2.1 x 250 mm, Waters Corporation) and a stepped gradient from 3% to 54% solvent B over 70 min followed by an increase to 80% B for 3 min at a flow of 200 µL/min. Solvent A was 25 mM ammonia and solvent B was 84% acetonitrile. 96 primary fractions were combined into 36 final fractions which were evaporated and reconstituted in 3% acetonitrile, 0.1% trifluoroacetic, 0.015% N-dodecyl-β-D-maltoside for LC-MS3 analysis.

LC-MS3 analysis

The fractions were analysed on an Orbitrap Eclipse™ Tribrid™ mass spectrometer equipped with a FAIMS Pro ion mobility system and interfaced with an Easy-nLC1200 liquid chromatography system (Thermo Fisher Scientific). Peptides were trapped on an Acclaim Pepmap 100 C18 trap column (100 µm x 2 cm, particle size 5 µm, Thermo Fisher Scientific) and separated on an in-house packed analytical column (35 cm x 75 µm, particle size 3 µm, Reprosil-Pur C18, Dr. Maisch) using a stepped gradient from 4% to 28% acetonitrile in 0.2% formic acid over 77 min at a flow of 300 nL/min. FAIMS Pro was alternating between the compensation voltages (CV) of -50 and -70, and the same data-dependent settings were used at both CVs. The precursor ion mass spectra were acquired at a resolution of 120 000 and an m/z range of 375-1500. Using a cycle time of 1.5 seconds the most abundant precursors with charges 2–7 were isolated with an m/z window of 0.7 and fragmented by collision-induced dissociation (CID) at 35%. Fragment spectra were recorded in the ion trap at Rapid scan rate. Dynamic exclusion was set to 60 sec. The ten most abundant MS2 fragment ions were isolated using multi-notch isolation for further MS3 fragmentation. MS3 fragmentation was performed using higher-energy collision dissociation (HCD) at 55% and the MS3 spectra were recorded in the Orbitrap at a resolution of 50 000 and an m/z range of 100–500.

Proteomic data analysis

Raw files were processed and analyzed with Proteome Discoverer (ver 3.0, Thermo Fisher Scientific). The data was matched against *Homo sapiens* SwissProt database (20597 entries, Feb 2024) using Sequest as a search engine with a precursor tolerance of 5 ppm and a fragment ion tolerance of 0.6 Da. Tryptic peptides were accepted with 1 missed cleavage. Methionine oxidation was set as a variable modification and cysteine carbamidomethylation, TMTpro on lysine and peptide N-termini were set as fixed modifications. A percolator was used for PSM validation with a strict FDR threshold of 1%. For quantification, TMT reporter ions were identified in the MS3 HCD spectra with 3 mmu mass tolerance and the TMT reporter intensity values for each sample were normalized on the total peptide amount. The SPS threshold was set to 65%, and a Sequest HT threshold score of 2 was chosen. Unique peptides were used for relative quantification and proteins were required to pass a protein FDR of 5%.
